# Experiences of discrimination and positive treatment among people who support a person with a mental health condition: a nationally representative survey

**DOI:** 10.1007/s00127-026-03137-w

**Published:** 2026-06-09

**Authors:** Christopher Rainbow, Amy J Morgan, Rachel Green, Gayle McNaught, Nicola J Reavley

**Affiliations:** 1 SANE, Melbourne, Australia; 2 Centre for Mental Health and Community Wellbeing, Melbourne School of Population and Global Health, The University of Melbourne, Victoria Melbourne, Australia

**Keywords:** Stigma, Discrimination, Caregivers, Caregiver support, Psychological distress, Wellbeing

## Abstract

**Purpose:**

Stigma and discrimination compound the burden experienced by informal caregivers of people with mental illness, impacting their wellbeing. Prior caregiver stigma research has relied on qualitative or convenience samples, limiting knowledge of groups most affected. This Australian study is the first nationally representative survey of caregiver experiences of discrimination and positive treatment.

**Methods:**

Participants (*N* = 2,164) were recruited from a probability-based online panel using stratified random sampling and weighting for age, gender, education, and language background. Descriptive statistics examined prevalence of stigma, discrimination and positive treatment experiences. Multiple regression models were used to assess sociodemographic differences in experiences and associations with psychological distress and wellbeing.

**Results:**

Positive treatment experiences were common, with 67.9% (95% CI: 65.4, 70.2) of caregivers reporting understanding from friends and family and 59.6% (95% CI: 57.1, 62.1) feeling valued by the care recipient’s treating team. However, 34.3% (95% CI: 31.9, 36.8) reported role concealment, and 18.8% (95% CI: 16.8, 20.9) unfavourable treatment at least sometimes. Positive treatment experiences were more prevalent among caregivers who were older or with higher levels of education, whereas experiences of discrimination were more common among caregivers from non-English speaking backgrounds. Women were less likely to conceal their caregiving role. Positive treatment was associated with lower distress and higher wellbeing, whereas discrimination was associated with higher distress and lower wellbeing.

**Conclusion:**

Despite encouraging levels of support, experiences of caregiver discrimination are common and impact on psychological distress and wellbeing. Multi-pronged strategies are needed to address inequities, strengthen peer support and caregiver-inclusive practice frameworks.

**Supplementary Information:**

The online version contains supplementary material available at https://doi.org/10.1007/s00127-026-03137-w.

## Introduction

Informal caregiving roles undertaken by family and friends are often the connective tissue in fragmented mental healthcare systems, encompassing a wide range of emotional, practical, and advocacy supports for people with mental health conditions [1, 2]. Caregivers themselves may require support across multiple domains, including emotional support, respite, financial assistance, access to services, and help navigating complex care systems [3]. In Australia, around one million people living with psychosocial disabilities such as mental health conditions receive informal assistance with at least one activity of daily life, most commonly from a parent, partner or child [4]. 1 The intensity of this care is often substantial, with weekly hours comparable to full-time employment [7]. Time is spent on both caregiving and in navigating overburdened mental health systems, which can be particularly challenging for those with complex or ongoing needs [8]. In addition to the frequently undervalued burden of care [9], studies across a range of cultural and national contexts have found that informal caregivers may face stigmatising attitudes and discrimination related to their support role or the mental health condition of the person they care for [10]. This is sometimes referred to as courtesy stigma or associative stigma, with research showing that such experiences are more common among those who have close or multiple relationships with people living with mental health conditions [11]. Unfavourable treatment by others such as lack of understanding, avoidance of mental health topics, blame, accusations of neglect or abuse, and exclusion by health professionals from a family member’s care are commonly reported [12–14]. Such experiences, or anticipation of them, can lead caregivers to conceal their role to avoid judgment [15]. Concealment adds to feelings of social isolation, exclusion and loneliness among caregivers [16] and can also delay professional help-seeking [17], impacting the mental health of both the family member and the caregiver.

These experiences may be more acute among caregivers supporting people with more severe or highly stigmatised conditions, such as schizophrenia [18]. A meta-synthesis of 16 studies on schizophrenia caregiving found that anticipated stigma and discrimination often compounded one another, with negative interactions involving employers, clinicians, and family members heightening caregiver distress and hypersensitivity, leading them to perceive stigma or negative treatment even in neutral encounters [19]. Stigma is also associated with poorer mental health and quality of life for caregivers. The caregiving role itself is often emotionally demanding and associated with unmet support needs, characterised by persistent worry, disruption of employment and relationships, financial strain, and repeated cycles of hope and despair [3, 16, 20–21]. Longitudinal evidence from an Australian population cohort study found that, over a two-year period, caregivers who were simultaneously managing full-time employment experienced declines in both physical functioning and mental health, whereas matched non-caregivers showed no significant change [22]. Stigma accentuates this burden, contributing to depression, anxiety, social withdrawal, loneliness, and reduced self-care [16, 20, 23]. Even when caregivers use effective coping strategies to manage burden, those who feel stigmatised are more likely to doubt their abilities or feel less in control of their situation [20]. These patterns have been documented across a wide range of international settings, suggesting that caregiver stigma is a common feature of mental health caregiving across diverse cultural contexts [19, 21, 23].

There is some evidence that the impacts of caregiver stigma may differ across sociodemographic groups, although direct comparisons are limited. In collectivist cultures, where mental illness may be seen as reflecting on the family [24], anticipated stigma can be intensified, leading caregivers to conceal their family member’s illness and withdraw socially [19, 25]. In countries where legal protections against discrimination are limited, families may face lower pay or outright job refusal from employers [19]. Some families in such countries report receiving blame from health professionals for their loved one’s condition, or having their loved one rejected by a spouse and returned to the family as defective [19]. In Western countries, young caregivers may also disengage from peers or avoid bringing friends home due to fear of judgment [26]. Some studies have found that caregivers with lower education levels report higher stress, greater perceived stigma, and lower social support [16, 27]. Gender-related findings are mixed, with some studies reporting greater unfair treatment directed toward female caregivers [28, 29], and others finding no differences [30].

Despite consistent evidence of the impact of stigma and discrimination on caregivers, existing larger-scale studies have typically relied on convenience samples recruited through mental health services or advocacy organisations [31–33]. As such, there remains a lack of representative national data to guide service and policy responses. In Australia, while studies have examined the economic contributions and needs of caregivers [3, 7], little is known about the actual prevalence of caregiver stigma, its demographic patterns, or associations with caregiver wellbeing. Addressing these gaps can help identify priority areas for support and guide the development of stigma-reduction interventions. The aims of this study were to carry out a population survey on caregiver experiences of discrimination and positive treatment (i.e., supportive or validating responses from others, including health professionals), the sociodemographic characteristics associated with these experiences and any associations with psychological distress and wellbeing.

## Method

### 2024-25 national survey of stigma and discrimination

The *2024-25 National Survey of Stigma and Discrimination* was a nationally representative survey designed to gather evidence on stigma and discrimination associated with mental health conditions. The survey was conducted as a partnership between SANE Australia, an Australian mental health service and advocacy organisation, and the University of Melbourne. Questions covered stigmatising attitudes, self-stigma, experiences of discrimination, positive treatment, and exposure to media reporting of mental ill health, the findings of which are reported elsewhere [34]. All survey questions underwent review by SANE’s Lived Experience Advisory Committee, which comprises representation from a diverse range of mental health consumers and caregivers. The current study focuses on a subset of questions targeted at caregiver respondents. This study was approved by the University of Melbourne Human Research Ethics Committee (ID30886).

### Participants and procedure

Data analysed in the current study comprises a weighted sample of caregivers drawn from 6,032 total participants in the full survey, completed in November and December 2024. Of this total sample, 35.1% (95% CI: 33.7, 36.3) reported caring for someone who had experienced a mental health problem in the previous 12 months (*N* = 2,164). Participants were recruited by the Social Research Centre from the Life in Australia™ probability-based panel. The panel is designed to provide a representative cross-section of Australian adults and was recruited using dual-frame landline and mobile random digit dialling. Most panellists complete surveys online; however, those who are unable or unwilling to complete surveys online are interviewed by telephone to ensure representation of the offline population [35]. Panellists were selected using stratified random sampling, with strata defined by age (18–34, 35–44, 45–54, 55–64, 65+), gender, education level, and home language use (English vs. other language). Survey invitations were distributed by email and SMS, with non-responders receiving up to five reminders throughout the data collection window. Reimbursement for participation was offered at AUD $10 or $20, depending on the length of the survey, and could be received as either a gift card or donated to a nominated charity. The overall survey completion rate, defined as the proportion of Life in Australia™ adult panel members invited to participate who completed the survey, was 63.7%. Demographics of the caregiver sample used is displayed in Table 1.

### Measures

#### Demographics

Participants were asked about state of residence, regionality (urban vs. regional), age, gender, Indigenous identity, country of birth, highest level of education completed, and whether they had experienced a mental health problem in the past 12 months (yes/no). Cultural and linguistic background was assessed using country of birth (categorised as primarily English-speaking vs. non-English-speaking countries) and whether English was the primary language spoken at home. These measures are widely used in Australian research as proxy indicators for culturally and linguistically diverse populations [36].

#### Caregiver status

Participants were asked if they had been a support person for someone who has experienced a mental health problem within the past 12 months (yes/no). For clarity, an explanation was provided alongside the question defining support person as someone who provides emotional or practical support for a parent, child, partner, friend, work colleague or other important person in the participant’s life.

#### Stigma, discrimination and positive treatment

**Fig. 1:**
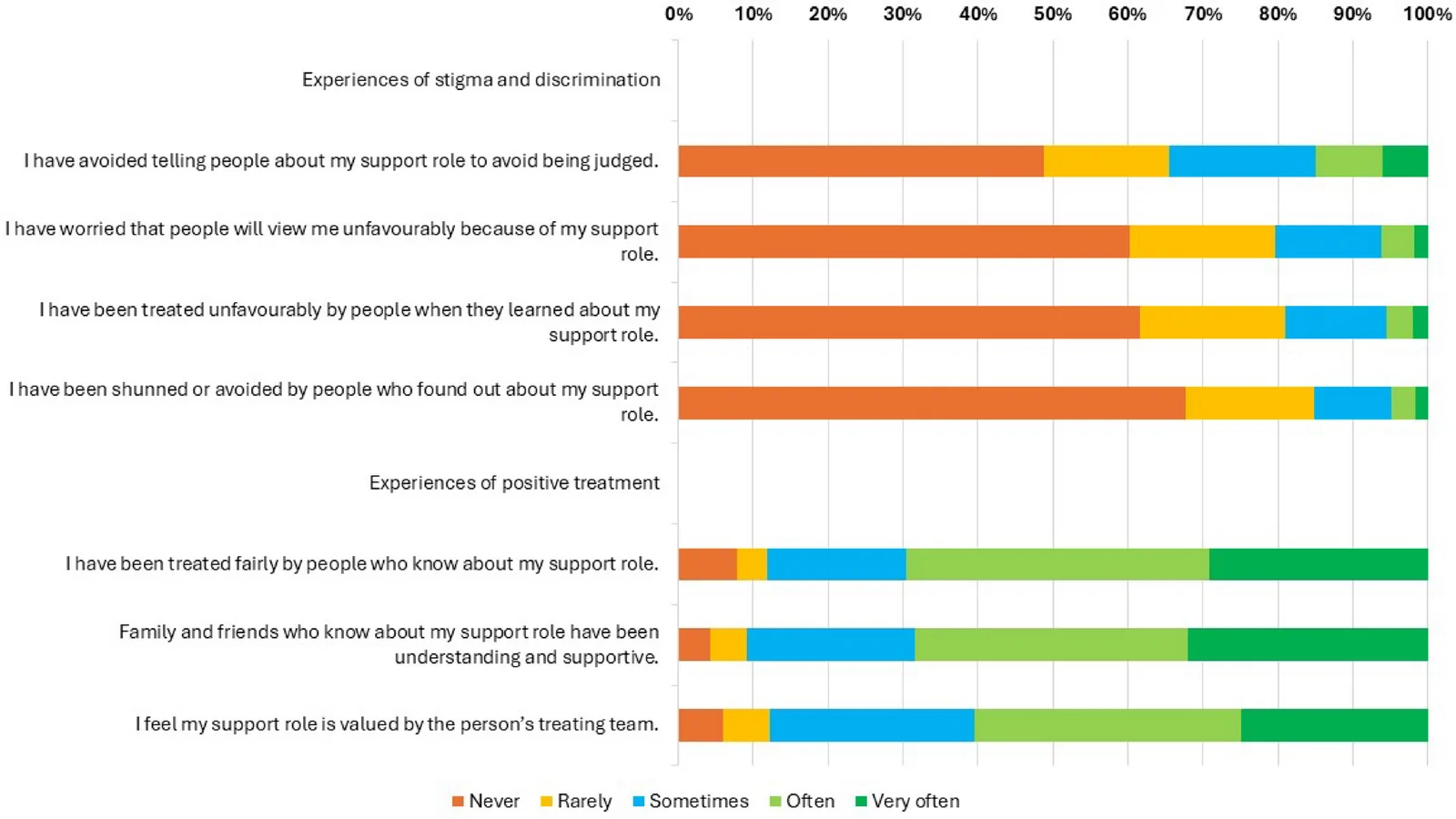
Prevalence of stigma, discrimination and positive treatment

Caregiver participants were displayed a series of seven statements (see Fig. 1) encompassing experiences of discrimination, anticipated stigma, and positive treatment received from family, friends or health professionals. A 5-point response scale from “never” to “very often” was used, with respondents selecting the category that best reflected the perceived frequency of each experience. No numerical thresholds were specified for the categories. Statements were derived from a prior Australian national survey, designed by the Mental Health Council of Australia with consumer and caregiver input [12].

#### Psychological distress

The short form of the Kessler Psychological Distress Scale (K6) measures anxious and depressive symptoms experienced over the past four weeks [37]. Across six items, participants report how often they have experienced various symptoms (e.g., restlessness, hopelessness) on a 5-point response scale from “none of the time” (1) to “all of the time” (5). Items are totalled, with scores ≥ 19 indicative of the likely presence of a mood disorder [38]. Internal consistency in the current sample was good (ω = 0.94).

#### Wellbeing

The 14-item Warwick-Edinburgh Mental Wellbeing Scale measures positive aspects of mental health experienced over the past two weeks [39]. Questions cover a wide range of feelings including optimism, usefulness, interest in other people and activities, belonging, energy, autonomy and self-confidence. Participants report how often they have experienced these feelings on a 5-point response scale from “none of the time” (1) to all of the time (4). Scores are totalled, with higher scores representing higher levels of positive affect and functioning. Internal consistency in the current sample was good (ω = 0.92).

### Weighting and analysis

To produce representative estimates for analysis, weighting was carried out through a two-stage process. First, a base weight was calculated for each respondent using three components: (1) an enrolment weight representing the chance of joining the panel and adjusted to demographic benchmarks; (2) a sample selection weight reflecting survey inclusion probability; and (3) a response propensity weight calculated from enrolment data for both respondents and non-respondents. Second, this base weight was adjusted to national population benchmarks including age, gender, education, language spoken at home, and geographic location taken from the 2021 Australian Census [40], and household adult count taken from the 2020-21 National Health Survey [41]. Prevalence of stigma, discrimination and positive treatment experiences were calculated using percentage frequencies and 95% confidence intervals. Regression analyses were used to explore associations between sociodemographic characteristics and endorsement of stigma and support experiences, as well as their association with psychological distress and wellbeing (controlling for sociodemographic characteristics). All analyses were performed using StataSE 18 [42].

## Results

### Prevalence of stigma, discrimination and positive treatment

Figure 1 gives the proportions of caregivers who reported experiences of discrimination in key domains as well as the frequency of experiences (full 95% CIs are supplied in Supplementary Table S1). The most commonly reported experience of anticipated stigma was caregiving role concealment due to fear of judgment, with 34.3% (95% CI: 31.9, 36.8) of participants endorsing concealment of their support role at least sometimes. The most commonly reported experience of discrimination was unfavourable treatment by people who learned about participants’ caregiving roles, with 18.8% (95% CI: 16.8, 20.9) reporting that this had occurred at least sometimes. Positive treatment was endorsed by the majority of participants often or very often, including family and friends being understanding (67.8%, 95% CI: 65.4, 70.2), and feeling valued by their person’s treating team (59.6%, 95% CI: 57.1, 62.1).

### Endorsement of stigma, discrimination and positive treatment by sociodemographic characteristics

Table 2 displays multiple linear regressions predicting endorsement of stigma, discrimination and positive treatment, controlling for sociodemographic characteristics. Older people (65+) were significantly less likely to report being shunned or avoided by people who found out about their caregiving role, be worried about receiving unfavourable treatment, and more likely to have received fair treatment. Women were significantly less likely to conceal their caregiving roles. Participants with higher levels of education were more likely to report fair treatment from others and receiving support from friends and family. Participants born in non-English speaking countries were more likely to report all forms of stigma and discrimination: role concealment, being shunned or avoided by people due to their caregiving role, fear of receiving and actually receiving unfavourable treatment. Non-English speaking background participants were also less likely to have experienced fair treatment from others.

### Associations between stigma, discrimination, positive treatment, psychological distress and wellbeing

Table 3 displays multiple linear regressions predicting psychological distress (K6) and wellbeing (WEMWBS) scores from stigma, discrimination and positive treatment. Significant associations were found between psychological distress, wellbeing and all experiences. Experiences of actual and anticipated discrimination were positively associated with psychological distress and negatively associated with wellbeing. Experiences of positive treatment were negatively associated with psychological distress and positively associated with wellbeing. Association effect sizes were all small to moderate, with the largest effects found between wellbeing, shunning and fear of unfair treatment (negative), family support and fair treatment (positive).

## Discussion

This study is the first nationally representative survey in Australia to examine both supportive and stigmatising experiences among mental health caregivers. Findings present a mixed picture: while many caregivers reported positive treatment, substantial proportions described role concealment, exclusion, and unfavourable treatment. These patterns varied across sociodemographic groups, suggesting that experiences may be shaped by age, gender, education, and cultural background. Older caregivers were less likely to report shunning, whereas caregivers born in non-English speaking countries were more likely to experience it. Women were less likely to report role concealment, and participants with higher education levels were more likely to report experiencing support from friends and family for their caregiving role. Although causal relationships cannot be established, caregivers reporting positive treatment were more likely to experience higher wellbeing and lower distress, while stigma and discrimination experiences were associated with lower wellbeing and higher distress.

### Experiences of stigma, discrimination and positive treatment

Positive treatment experiences were common, particularly feeling understood by friends and family and valued by practitioners. Recognition of the caregiving role can enhance caregivers’ sense of value and connection, and may also benefit care recipients [43]. One large previous Australian survey, from which the current study’s stigma statements were derived, reported lower levels of support from health professionals [12]. Convenience sampling in this survey may have resulted in a pool of participants with a higher prevalence of negative experiences, but the higher rates of positive treatment observed in the current study may also reflect health system shifts towards carer-inclusive models of care, although these are inconsistently implemented [44]. Greater understanding from friends and family may also be influenced by national mental health awareness campaigns, which have improved general attitudes toward mental illness, although less so for more complex conditions such as schizophrenia [45]. Despite this, nearly one in five participants reported unfavourable treatment due to their caregiving role and one in three had concealed their role. These results align with extensive international literature across multiple continents showing that internalised stigma and anticipated rejection are common [46], may contribute to social withdrawal [16], reduced help-seeking [17], and increased distress [23].

### Sociodemographic patterns

Experiences varied across groups. Older caregivers (65+) were less likely than younger caregivers (18–34) to report being shunned or avoided, although this should be interpreted alongside the broader challenges faced by many older carers, including poorer health, limited formal support, and financial strain due to a higher likelihood of living on a fixed income or no longer being in the workforce [47]. Younger caregivers may experience greater social exclusion due to a perceived mismatch between their life stage and caregiving responsibilities [26]. Gender differences were also evident. Women were less likely than men to conceal their caregiving role, potentially reflecting cultural expectations that position caregiving as a predominantly female responsibility [48]. Masculine norms of self-reliance [49] may make men less likely to disclose caregiving responsibilities, with self-reliance also linked to lower help-seeking and higher self-stigma [50, 51].

Participants with higher education levels more often reported understanding from friends and family and fair treatment by others. In contrast, participants born in non-English speaking countries were more likely to experience shunning or avoidance and less likely to report fair treatment. This is consistent with prior studies that have found an association between lower education levels, and higher stress and perceived stigma among caregivers [16, 27]. Education is closely tied to socioeconomic privilege, which can buffer against depression [52] and provide access to more responsive service environments [53]. For non-English speaking background caregivers, while prior research has noted that stigma may be intensified by cultural differences in the conceptualisation of mental illness [24], living in the context of an English-speaking Western country like Australia should also be considered: service access and discrimination may be complicated by language barriers [54], and broader experiences of racism and exclusion [55].

### Associations with psychological distress and wellbeing

Consistent with previous studies [14, 19, 46], experiences of stigma and discrimination in this study were associated with higher distress and lower wellbeing, with the strongest effects observed for being shunned and for worry about being viewed unfavourably. Prior research suggests these associations may be bidirectional, with stigma contributing to poorer mental health, which may in turn heighten perceptions of stigma [56]. Supportive experiences, including fair treatment, family understanding, and feeling valued by practitioners, were linked with higher wellbeing, although their associations with distress were comparatively weaker. This may reflect the fact that support and inclusion can provide hope for the care recipient’s future [12, 14], but does not necessarily ease the daily burden of caregiving [20, 21]. Indeed, more than half of all participants reported experiencing a mental health problem themselves in the past year, and more than one-third were experiencing clinically significant levels of psychological distress, levels much higher than the general Australian population [57]. Although a range of services and support programmes are available for caregivers in Australia, many still report unmet support needs [3]. The current study did not assess whether caregivers sought professional support for their own distress. Future research should examine help-seeking among distressed caregivers, including potential barriers such as time constraints, limited health professional recognition of caregiver needs, and stigma related to accessing mental health care.

### Implications for practice and policy

The results underscore the importance of addressing the harmful impacts of stigma while promoting inclusion and recognition. While anti-stigma campaigns can be effective [58], their impact is often modest [59] and may fail to target high-stigma groups or reflect community diversity [60]. A broader approach addressing structural and systemic drivers may prove more fruitful. For non-English speaking background caregivers, this may include considering cultural understandings of mental illness alongside racism, language barriers, and inequitable access to services [54, 55]. It is important to note that not all non-English speaking background caregivers' experiences are homogenous, and our study is limited insofar as we were unable to fully explore how cultural nuances differentially shape experiences of stigma and discrimination. Future research in this area should explore the impacts on stigma of ethnicity, country of birth, and migration history to develop culturally tailored stigma reduction strategies. Nonetheless, our findings are consistent with a recent scoping review of 23 Australian studies finding that stigma and discrimination are most frequently reported as barriers to mental health service access in migrants from a range of non-English speaking background populations [61]. Similar targeted attention is warranted for male and younger caregivers, who in this study reported greater exposure to stigma. Online anonymous peer forums may help by enabling caregivers to connect without revealing their identity, normalising caregiving, reducing isolation, and building belonging [62]. Health professionals and services can play a central role in recognising and involving caregivers as contributors to care, while also supporting families to navigate the system by linking them with other appropriate community and peer networks [8]. Carer-inclusive practice frameworks exist in many jurisdictions but are inconsistently applied [44]. Training, supervision, and organisational accountability may help to support more consistent implementation.

### Strengths and limitations

This study provides the first nationally weighted estimates of stigma, discrimination and positive treatment experiences among Australian mental health caregivers. The representative sample and inclusion of demographic covariates enhance generalisability and allow examination of subgroup differences. Limitations include the cross-sectional design, which prevents causal inference, and a relatively small set of stigma-related items. Additionally, the survey was conducted through the Life in Australia™ probability-based panel. Interviews are conducted in English and recruitment relies on telephone sampling, which may underrepresent people with limited English proficiency and the small proportion of adults without telephone access [35]. Future research could involve the exploration of caregiver discrimination experiences in settings such as workplaces or schools. For example, caregiving needs are seen by some employers as a private problem rather than a social responsibility [63], which could lead to role concealment by the caregiver. While stigma in educational settings has been explored in students [64], caregiver stigma in schools is an under-researched area worthy of future investigation. Future research could also explore the prevalence and impact of witnessing stigma towards a loved one, which can add to caregiver experiences of psychological distress [10]. The survey did not collect diagnostic information about care recipients, which could influence stigma severity, particularly for highly stigmatised conditions such as schizophrenia, bipolar and personality disorders [60]. Future research could investigate diagnosis-specific experiences and track the longitudinal effects of stigma, discrimination and positive treatment.

### Conclusion

This survey offers new insights into the complex experiences of mental health caregiving in Australia. Many caregivers reported feeling understood by friends and family and valued by practitioners, forms of recognition that may help sustain their role. At the same time, a significant minority experienced exclusion or concealed their role, particularly younger, male, and non-English speaking background caregivers. Associations between exclusion, concealment, and poorer wellbeing point to the potential psychological toll of stigma, while positive associations between wellbeing and positive treatment highlight potential benefits of inclusion. Embedding carer-inclusive practice into routine care, supported by training and accountability measures, will be important for ensuring caregivers are recognised as essential partners in mental health support systems. Strategies that target high-stigma groups, address structural barriers, and reflect the diversity of caregivers may help to reduce stigma, support wellbeing, and fully recognise the undervalued burden of informal caregiving for mental illness.

**Table 1:** Sample characteristics

	*n*	% (95% CI)
*Gender*		
Male	684	41.2 (38.6, 43.8)
Female	1449	57.0 (54.4, 59.5)
Prefers another term	30	1.8 (1.2, 2.8)
*Age groups*		
18–34	427	31.2 (28.6, 33.9)
34–64	1367	53.6 (51.0, 56.2)
65+	369	15.2 (13.6, 16.9)
*Indigenous identity*		
Aboriginal and/or Torres Strait Islander	70	3.9 (3.0, 5.1)
Non-Indigenous	2085	96.1 (94.9, 97.0)
*Language spoken at home*		
English	1851	78.9 (76.5, 81.2)
Other	313	21.1 (18.8, 23.5)
*Country of birth*		
English speaking	1871	84.2 (82.1, 86.0)
Non-English speaking	291	15.8 (14.0, 17.9)
*Level of education*		
Below Bachelor	1050	68.1 (65.9, 70.2)
Bachelor or above	1099	31.9 (29.8, 34.1)
*Psychological distress*		
*K6*		
K6 score *≥* 19	705	35.9 (33.5, 38.5)
K6 score *≤* 18	1459	64.1 (61.5, 66.5)
*Personal experience of a mental health problem in the last 12 months*		
Yes	1116	53.1 (50.5, 55.6)
No	1048	46.9 (44.4, 49.5)

**Table 2:** Multiple linear regressions predicting endorsement of stigma, discrimination and positive treatment items by sociodemographic characteristics

Item	Age (ref 18–34)		Gender (ref male)		Education (ref ≤ High school)	Country of birth (ref main English speaking)
	35–64 years	65 + years	Female	Other	≥ Bachelor	Non-English speaking
*Experiences of stigma and discrimination*						
I have avoided telling people about my support role to avoid being judged.	0.09 [-0.08,0.26]	-0.09 [-0.29,0.12]	-0.14 [-0.28, -0.00]*	0.18 [-0.41, 0.76]	0.04 [-0.10, 0.17]	0.25 [0.04, 0.45]*
I have worried that people will view me unfavourably because of my support role.	0.00 [-0.13,0.14]	-0.25 [-0.41, -0.10]**	0.04 [-0.07, 0.14]	-0.07 [-0.43, 0.29]	0.00 [-0.10, 0.11]	0.19 [0.03, 0.35]*
I have been treated unfavourably by people when they learned about my support role.	0.03 [-0.10,0.17]	-0.16 [-0.32, 0.00]	0.03 [-0.08, 0.14]	0.07 [-0.33, 0.46]	-0.10 [-0.21, 0.01]	0.29 [0.10, 0.47]**
I have been shunned or avoided by people who found out about my support role.	-0.07 [-0.20,0.07]	-0.29 [-0.43, -0.15]**	-0.01 [-0.12, 0.09]	-0.26 [-0.52, 0.00]	-0.10 [-0.20, 0.00]	0.38 [0.20, 0.56]**
*Experiences of positive treatment*						
I have been treated fairly by people who know about my support role.	0.05 [-0.11,0.21]	0.20 [0.01, 0.40]*	0.00 [-0.13, 0.13]	-0.12 [-0.66, 0.43]	0.15 [0.02, 0.27]*	-0.18 [-0.36, -0.00]*
Family and friends who know about my support role have been understanding and supportive.	0.00 [-0.14,0.14]	0.15 [-0.03, 0.32]	0.01 [-0.10, 0.12]	0.05 [-0.33, 0.43]	0.17 [0.06, 0.27]**	-0.11 [-0.27,0.04]
I feel my support role is valued by the person’s treating team.	-0.10 [-0.23, 0.03]	-0.10 [-0.28, 0.08]	-0.04 [-0.16, 0.07]	-0.08 [-0.55, 0.38]	-0.02 [-0.13, 0.09]	0.04 [-0.11, 0.19]

*Notes.* Values are unstandardised regression coefficients (B) with 95% confidence intervals in brackets. All outcomes are continuous endorsement scores for individual stigma/support items. * *p* < .05. ** *p* < .01

**Table 3:** Multiple linear regressions predicting psychological distress and wellbeing from stigma and support experiences

Item	Psychological distress (K6)		Wellbeing (WEMWBS)	
	B [95% CI]	β	B [95% CI]	β
*Experiences of stigma and discrimination*				
I have avoided telling people about my support role to avoid being judged.	0.75 [0.49,1.02]	0.15	-0.66 [-0.89,-0.44]	-0.17
I have worried that people will view me unfavourably because of my support role.	1.30 [0.96,1.64]	0.20	-1.14 [-1.44,-0.83]	-0.22
I have been treated unfavourably by people when they learned about my support role.	1.09 [0.73,1.46]	0.17	-0.86 [-1.20,-0.52]	-0.16
I have been shunned or avoided by people who found out about my support role.	1.40 [1.04,1.76]	0.20	-1.17 [-1.51,-0.84]	-0.21
*Experiences of positive treatment*				
I have been treated fairly by people who know about my support role.	-0.70 [-0.99,-0.40]	-0.13	1.01 [0.75,1.26]	0.23
Family/friends who know about my support role have been understanding and supportive.	-0.84 [-1.15,-0.53]	-0.14	1.25 [0.98,1.52]	0.26
I feel my support role is valued by the person’s treating team.	-0.39 [-0.67,-0.11]	-0.07	0.85 [0.60,1.10]	0.18

Note. All outcomes are continuous endorsement scores for psychological distress (K6) and wellbeing (WEMWBS). All regressions controlled for age, gender, education and country of birth. All effects are significant at *p* < .001

## Electronic Supplementary Material

Below is the link to the electronic supplementary material.

Supplementary material 1

## Data Availability

Data available upon reasonable request from the authors.

## Footnotes

1 The authors recognise and understand that the terminology used to describe people who provide unpaid support to someone experiencing mental health conditions is contested. Terms such as carer, caregiver, and family supporter are used inconsistently across research, policy, and lived-experience contexts, and many individuals who provide substantial assistance do not necessarily identify with these labels [5]. For consistency, this paper uses the terms caregiver and informal caregiver to refer to any person with a recent lived experience of providing voluntary personal care, support, advocacy, and/or assistance for a person with a mental health condition in an ongoing capacity [6].
