# Supplementary material for: Experiences of discrimination and positive treatment among people who support a person with a mental health condition: a nationally representative survey
Source: Soc Psychiatry Psychiatr Epidemiol. 2026 Jun 9;61(11):2313–23. doi: 10.1007/s00127-026-03137-w (PMC13694312; doi:10.1007/s00127-026-03137-w)
Supplement: Supplementary file 1 [file 127_2026_3137_MOESM1_ESM.docx]

## Supplementary Table S1. *Prevalence of stigma, discrimination and positive treatment experiences.*

| Item | Never | Rarely | Sometimes | Often | Very often |
| --- | --- | --- | --- | --- | --- |
| Experiences of stigma and discrimination |  |  |  |  |  |
| I have avoided telling people about my support role to avoid being judged. | 48.6 [46, 51.1] | 16.6 [14.8, 18.6] | 19.3 [17.3, 21.4] | 8.9 [7.6, 10.4] | 6.1 [4.9, 7.6] |
| I have worried that people will view me unfavourably because of my support role. | 59.8 [57.2, 62.3] | 19.3 [17.3, 21.4] | 14.0 [12.3, 15.9] | 4.3 [3.3, 5.5] | 1.9 [1.3, 2.7] |
| I have been treated unfavourably by people when they learned about my support role. | 60.9 [58.4, 63.4] | 19.1 [17.2, 21.2] | 13.3 [11.6, 15.2] | 3.5 [2.6, 4.6] | 2.0 [1.3, 3.0] |
| I have been shunned or avoided by people who found out about my support role. | 67.0 [64.6, 69.4] | 17.0 [15.2, 19.0] | 10.3 [8.8, 12.0] | 3.2 [2.3, 4.3] | 1.6 [1.0, 2.5] |
| Experiences of positive treatment |  |  |  |  |  |
| I have been treated fairly by people who know about my support role. | 7.7 [6.4, 9.3] | 4.1 [3.2, 5.3] | 18.3 [16.4, 20.4] | 40 [37.5, 42.5] | 28.9 [26.6, 31.3] |
| Family and friends who know about my support role have been understanding and supportive. | 4.3 [3.3, 5.4] | 4.8 [3.8, 6] | 22.2 [20.1, 24.4] | 36.1 [33.6, 38.6] | 31.8 [29.4, 34.2] |
| I feel my support role is valued by the person’s treating team. | 5.9 [4.9, 7.2] | 6.2 [5.1, 7.5] | 26.9 [24.6, 29.2] | 35 [32.5, 37.4] | 24.6 [22.5, 26.9] |

*Note.* Values are weighted percentages with 95% confidence intervals.
